# Increasing global agricultural production by reducing ozone damages via methane emission controls and ozone-resistant cultivar selection

**DOI:** 10.1111/gcb.12118

**Published:** 2013-02-05

**Authors:** Shiri Avnery, Denise L Mauzerall, Arlene M Fiore

**Affiliations:** * Program in Science, Technology, and Environmental Policy, Woodrow Wilson School of Public and International Affairs, Princeton UniversityPrinceton, NJ, 08544, USA; † Department of Civil and Environmental Engineering, Princeton UniversityPrinceton, NJ, 08544, USA; ‡ Geophysical Fluid Dynamics Laboratory, National Oceanic and Atmospheric Administration, Princeton UniversityPrinceton, NJ, 08540, USA

**Keywords:** agriculture, crop sensitivity to O_3_, cultivar selection, methane mitigation, ozone impacts, surface ozone

## Abstract

Meeting the projected 50% increase in global grain demand by 2030 without further environmental degradation poses a major challenge for agricultural production. Because surface ozone (O_3_) has a significant negative impact on crop yields, one way to increase future production is to reduce O_3_-induced agricultural losses. We present two strategies whereby O_3_ damage to crops may be reduced. We first examine the potential benefits of an O_3_ mitigation strategy motivated by climate change goals: gradual emission reductions of methane (CH_4_), an important greenhouse gas and tropospheric O_3_ precursor that has not yet been targeted for O_3_ pollution abatement. Our second strategy focuses on adapting crops to O_3_ exposure by selecting cultivars with demonstrated O_3_ resistance. We find that the CH_4_ reductions considered would increase global production of soybean, maize, and wheat by 23–102 Mt in 2030 – the equivalent of a ∼2–8% increase in year 2000 production worth $3.5–15 billion worldwide (USD_2000_), increasing the cost effectiveness of this CH_4_ mitigation policy. Choosing crop varieties with O_3_ resistance (relative to median-sensitivity cultivars) could improve global agricultural production in 2030 by over 140 Mt, the equivalent of a 12% increase in 2000 production worth ∼$22 billion. Benefits are dominated by improvements for wheat in South Asia, where O_3_-induced crop losses would otherwise be severe. Combining the two strategies generates benefits that are less than fully additive, given the nature of O_3_ effects on crops. Our results demonstrate the significant potential to sustainably improve global agricultural production by decreasing O_3_-induced reductions in crop yields.

## Introduction

From 2010 to 2030 the demand for grain is expected to increase globally by 50% (Food & Agriculture Organization of the United Nations, 2006; World Bank, 2007) due to an increase in global population of roughly 1.4 billion people (US Census Bureau, 2010), a shift to a more diverse, animal protein-rich diet associated with rising living standards, and the expansion of global biofuel production. Agricultural production has historically kept pace with surging demand primarily by improving yields on existing farmland through increasing water, fertilizer, and pesticide application and employing other technologies associated with the Green Revolution (Burney *et al*., 2010). However, the prospects for meeting future global grain demand via agricultural intensification (i.e., yield improvements) on land already under cultivation remain uncertain. The yield growth rates of some key staple crops have been stagnant or declining over the last few decades in many parts of the world, especially in South and East Asia (Tilman *et al*., 2002; World Bank, 2007; Dasgupta & Sirohi, 2010). In the absence of yield improvements, meeting the rising global food demand of the future will likely require an increase in farmland area – leading to the loss of biodiversity and potentially tremendous emissions of carbon. For example, recent work estimates that without the historic yield increases of the past half century, present-day agricultural demand would have required cropland expansion of over 1700 million hectares, an area greater than the ∼1500–1600 million hectares under cultivation today (Lambin & Meyfroidt, 2011), with resulting emissions of up to 161 gigatons of carbon (GtC, 1 GtC = 10^9^ metric tons) (Burney *et al*., 2010).

Although yield improvements are thus generally preferable to increasing crop production (CP) area from a biodiversity and a climate perspective, traditional means of agricultural intensification also have damaging environmental impacts associated with them from irrigation, chemical application, and other farming practices (Tilman *et al*., 2001, 2002; Gregory *et al*., 2002). As such, meeting the agricultural demand of over 8 billion people in 2030 without increasing environmental stress requires new approaches beyond cropland expansion and the traditionally employed portfolio of yield improvement strategies.

One way to improve agricultural production without negative environmental consequences is by reducing the damage – and associated yield reductions – caused by crop exposure to surface ozone (O_3_). O_3_ is a major component of smog and a potent greenhouse gas (GHG) produced in the troposphere by photochemical reactions between nitrogen oxides (NO_x_ = NO + NO_2_), carbon monoxide (CO), methane (CH_4_), and nonmethane volatile organic compounds (NMVOCs) (Forster *et al*., 2007). In addition to having a detrimental effect on human health (US Environmental Protection Agency, 1996; Bell *et al*., 2004; Jerrett *et al*., 2009), O_3_ has been found to be the air pollutant most damaging to vegetation (Heagle, 1989; Heck,1989), including crops. Recent studies estimate that the global yields of key staple crops are being reduced by 2–15% due to present-day ozone exposure (Feng & Kobayashi, 2009; Van Dingenen *et al*., 2009; Fishman *et al*., 2010; Avnery *et al*., 2011a Ozone-sensitive crops could see a further 10% decline in yields by 2030 if global O_3_ precursor emissions continue to increase (Van Dingenen *et al*., 2009; Avnery *et al*., 2011b Although O_3_ reductions via mitigation of conventional pollutant precursors (NO_x_, CO, and NMVOCs) would prevent significant additional future yield reductions (Van Dingenen *et al*., 2009; Avnery *et al*., 2011b even with aggressive emission controls global year 2030 losses could remain substantial – particularly for O_3_-sensitive crops (e.g., up to 17% globally for wheat with considerable regional variability) (Avnery *et al*., 2011b It is therefore worthwhile to explore supplemental strategies to reduce O_3_-induced crop losses beyond the targeting of traditional short-lived O_3_ precursors.

Here, we investigate two such supplemental strategies to decrease O_3_ damage to crops (soybean, maize, and wheat) and thereby improve agricultural yields. Our first strategy focuses on reducing surface O_3_ concentrations – and resultant crop exposure to O_3_ – via methane abatement (we hereafter refer to this scenario as our ‘mitigation’ strategy). CH_4_ is the second most important GHG after carbon dioxide (Forster *et al*., 2007) and has not previously been targeted for air quality purposes despite contributing to global background O_3_ concentrations (Fiore *et al*., 2002). However, decreases in CH_4_ emissions result in the greatest decrease in net radiative forcing per unit reduction in surface O_3_ of any O_3_ precursor (West *et al*., 2007). CH_4_ abatement therefore provides an attractive ‘win-win’ policy opportunity for both climate change and air pollution mitigation goals, as CH_4_ controls would reduce radiative forcing of climate while simultaneously achieving the health and agricultural benefits associated with surface O_3_ reductions (Shindell *et al*., 2012). Here, we quantify the CP improvements possible with a policy of methane controls (described in Section ‘MOZART-2 and model simulations’) relative to the ‘current legislation’ (CLE) emissions baseline. Under CLE, global anthropogenic CH_4_ emissions are projected to increase by 35% between 2000 and 2030 whereas existing legislation controlling the emissions of traditional air pollutants is assumed to be perfectly implemented (Dentener *et al*., 2005; Cofala *et al*., 2007).

The second strategy we explore to reduce O_3_-induced agricultural losses focuses on adapting crops to elevated levels of O_3_ via cultivar selection (we hereafter refer to this as our ‘adaptation’ policy). Large-scale, comprehensive field studies that took place primarily in the United States and Europe in the 1980s/1990s established the existence of a wide range of crop sensitivity to ozone, both among different crops and between cultivars of the same crop (Heagle, 1989; Heck, 1989; Krupa *et al*., 1998). Crop varieties used today appear to exhibit ozone sensitivity at least as great as that seen in earlier field studies (Long *et al*., 2005; Biswas *et al*., 2008; Emberson *et al*., 2009; Singh *et al*., 2010a,b; Zhu *et al*., 2011; Grünhage *et al*., 2012; Wang *et al*., 2012), suggesting that O_3_ sensitivity may be an overlooked factor in cultivar choice. To draw attention to this issue, we estimate the amount by which CP could potentially be improved by cultivating crop varieties with the greatest demonstrated O_3_ resistance (from large-scale US field studies (Heck *et al*., 2013; Heagle, 1989; Heck, 1989) relative to ‘median sensitivity’ cultivars under 2030 CLE (i.e., a future scenario where no new climate or ozone abatement measures are implemented over the next few decades).

Finally, we combine these two strategies to estimate the extent by which agricultural production could be improved by both CH_4_ emission controls and careful cultivar selection. We therefore explore two different strategies to reduce the detrimental impact of O_3_ on crops – one based on mitigating O_3_ concentrations and corresponding agricultural damages through controls on methane emissions, and one based on adapting crops to elevated levels of O_3_ exposure – and their combined effectiveness to demonstrate the potential of two complementary methods to improve global food production without further harm to the environment.

## Materials and methods

### MOZART-2 and model simulations

We use multidecadal full-chemistry transient simulations of the MOZART-2 global CTM (Horowitz et al., 2013) to project the response of surface O_3_ to future CH_4_ emissions from 2000 to 2030 under the CLE (Dentener *et al*., 2005; Cofala *et al*., 2007) and the reduced CH_4_ (CH_4_-red) scenarios, with the period 2000–2004 used for spin up (Fiore *et al*., 2008; the CH_4_-red scenario here corresponds to their scenario B). Simulations are driven by meteorological fields from the NCEP reanalysis (Kalnay *et al*., 1996) for 2000–2004, recycled every 5 years to allow for interannual variability in the O_3_ response to CH_4_, at 1.9° × 1.9° horizontal resolution with 28 vertical levels. In the CLE scenario, global anthropogenic emissions of CH_4_, NO_x_, CO, and NMVOC change by +29% (+96 Mt CH_4_ yr^−1^), +19% (+5.3 Mt N yr^−1^), −10% (−44 Mt CO yr^−1^), and +3% (+3 Mt C yr^−1^), respectively, from 2005 to 2030 (Dentener *et al*., 2005; Cofala *et al*., 2007; Fiore *et al*., 2008). In the CH_4_-red scenario, methane controls begin in 2006 and gradually increase to 125 Mt yr^−1^ by 2030 relative to the CLE baseline (along a near linear path before flattening out, with most reductions in place by 2020), representing nearly a 30% reduction in global anthropogenic year 2030 CH_4_ emissions (Fiore *et al*., 2008). The marginal cost of the methane reductions considered is estimated to be less than zero through 2017 rising to $161 per ton CH_4_ by 2030, with controls found to be cost effective given available technologies at a marginal cost of approximately $315 per ton CH_4_ ($15 per ton CO_2_ equivalent) (Fiore *et al*., 2008; West *et al*., 2012).

Anthropogenic CH_4_, defined as emissions originating from the agricultural and industrial sectors, contributes ∼0.7 Wm^−2^ to climate forcing (including O_3_ forcing) and 4 ppbv to surface O_3_ in year 2030 CLE (Fiore *et al*., 2008). The CLE and CH_4_-red simulations are transient (i.e., not in steady state), such that the full benefits of the gradually increasing CH_4_ reductions will not be realized by 2030 due to the relatively long lifetime of methane (∼12 years). The ‘effective CH_4_ emissions control’ in year 2030, which represents the change in CH_4_ emissions that would produce a steady-state response equal to the transient response in 2030, corresponds to 76 Mt CH_4_ yr^−1^, or ∼61% of the total CH_4_ emission reductions implemented by 2030 (Fiore *et al*., 2008). See Fiore *et al*. (2008) and the Supporting Information (SI) for an evaluation of the simulations used here.

### Reductions in surface ozone exposure due to methane mitigation

We calculate the difference (CLE − CH_4_-red) in year 2030 crop exposure to O_3_ using two biologically relevant metrics (AOT40 and W126) that policymakers in Europe and the United States, respectively, favor to set standards for the protection of sensitive vegetation. These two indices of O_3_ exposure (defined below, see SI for further discussion) were derived from large-scale field studies and characterize O_3_ exposure during crop growing seasons:


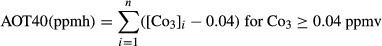



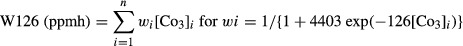


where:

- [Co_3_]_*i*_ is the hourly mean O_3_ concentration during local daylight hours (08:00–19:59); and
- *n* is the number of hours in the 3-month growing season (defined in Section Crop production and economic gains).

The AOT40 index was historically favored in Europe as the exposure-based metric that most accurately predicts the yield response of crops to O_3_. It is highly correlated with cumulative O_3_ exposure above a threshold of 40 ppbv (Krupa *et al*., 1998) and is based on the results of field studies conducted in the United States and Europe (Mills *et al*., 2007). The W126 function was derived from US field studies; it uses a sigmoidal function to assign greater weight to higher levels of hourly O_3_ concentrations with an inflection point at ∼65 ppbv (Lefohn & Runeckles, 1988). Although European ‘critical levels’ to protect crops and ecosystems have existed for over a decade, the most recent proposal to set a similar standard in the United States was recently withdrawn (as of September 2011) amid pressure from industry and business groups that argued new regulations would be too costly. However, the W126 metric remains favored by the US Environmental Protection Agency (EPA) and will likely continue to be the index proposed to serve as a secondary O_3_ standard in the next review of US O_3_ regulations (scheduled for 2013).

An important caveat about the exposure-based metrics used here and elsewhere to quantify O_3_-induced crop yield losses at large scales (Wang & Mauzerall, 2004; Van Dingenen *et al*., 2009; Avnery *et al*., 2011a,b; Hollaway *et al*., 2012; Shindell *et al*., 2012) is that they do not account for environmental factors that may moderate stomatal conductance (e.g., temperature, water availability, and CO_2_ concentrations), and therefore the actual flux of O_3_ into plants. Over a decade of research in Europe has led to the development of more biologically relevant models that simulate the flux of ozone through plant stomates using mathematical equations to characterize the species-specific impact of temperature, photosynthetic photon flux density, soil water potential, vapor pressure deficit, and plant growth stage on stomatal conductance (e.g., Pleijel *et al*., 2004; Mills *et al*., 2011a Maps of AOT40 exposure in Europe suggest significantly different spatial patterns of ozone risk to vegetation compared with those generated by flux models (Simpson *et al*., 2007), and observational evidence indicates a better match of actual O_3_ impacts with flux-based assessments (Mills *et al*., 2011b Given the greater accuracy of O_3_ flux models, Europe is moving toward a flux-based (rather than exposure-based) definition of critical levels, and has developed flux models for wheat, potato, tomato, and two tree species (beech and birch). However, further model specification and evaluation is required for additional crops and growing regions around the world; as such, flux-based indices are not yet suitable for regional or global impact analysis such as that performed here (Fuhrer, 2009).

### Crop production and economic gains

For each O_3_ exposure metric and crop cultivar, concentration : response (CR) relationships have been obtained by fitting linear, quadratic, or Weibull functions to the yields of crops grown under different levels of O_3_ during a 3-month growing season (Heagle, 1989; Heck, 1989; Lee & Hogsett, 1996; Krupa *et al*., 1998) (see SI for further details). Following previous studies (Van Dingenen *et al*., 2009; Avnery *et al*., 2011a,b), ‘growing season’ is defined here as the 3 months prior to the start of the harvest period in every country according to crop calendar data from the United States Department of Agriculture (USDA) (US Department of Agriculture, 1994, 2008) where data are available (accounting for over 95% of global production). The CR relationship for the AOT40 metric is linear, whereas the W126 index has a sigmoidal form following the shape of the weighting function (Fig. S1, Table S1). Because robust CR data are lacking for Asia, Africa, and South America, we apply the CR functions from the United States and Europe globally.

We follow previous studies (Wang & Mauzerall, 2004; Van Dingenen *et al*., 2009; Avnery *et al*., 2011a,b) and use CR functions representative of median or mean crop sensitivity to represent the baseline sensitivity of each crop to O_3_ (Table S1), as no single CR relationship can accurately represent the response of all crop cultivars grown worldwide. (In actuality, the total response of crops to ozone will be a weighted average of the responsiveness of each cultivar to its ozone exposure and its proportion of total acreage.) For the W126 metric, the EPA pooled US experiments and estimated parameter values across cultivars, locations, and years; it identifies a ‘median composite function’ that describes the 50th percentile response of crops, which we use as our baseline sensitivity for this metric (Lee & Hogsett, 1996). For AOT40, baseline sensitivity is the derived best-fit line generated from regression analysis of crop response to O_3_ concentrations (representing the mean crop response to O_3_) from field studies in both the United States and Europe (Mills *et al*., 2007).

To examine the benefits to agriculture of our methane mitigation policy, we compare crop production losses (CPL, discussed further below) as calculated under the CLE and CH_4_-red scenarios according to baseline (median or mean) sensitivity crop response to O_3_ (we refer to these scenarios as CLE_med_ and CH_4_-red_med_, respectively). We additionally examine the benefits to crops of adapting to high levels of O_3_ by choosing soybean, maize, and wheat cultivars with the greatest demonstrated ozone tolerance (i.e., minimum sensitivity varieties; Table S1), according to the W126 metric, compared with baseline cultivars in both the CLE and CH_4_-red scenarios (see SI). We refer to these cases as CLE_min_ and CH_4_-red_min_, respectively. We use only CR functions corresponding to the W126 metric to analyze the benefits of adaptation because, although individual cultivars demonstrated variability in O_3_ sensitivity, no statistically significant differences were found in the slopes of the regression lines of AOT40 CR functions (Mills *et al*., 2003, 2007). Our results should be considered illustrative (rather than a definitive estimate) of the potential benefits of cultivating crops with greater O_3_ tolerance given uncertainties in O_3_ sensitivity among cultivars grown around the world. However, our results are more than simply theoretical, as our analysis is based on the actual range of O_3_ sensitivity found among common cultivars in the comprehensive, large-scale US National Crop Loss Assessment Network (NCLAN) field studies (Heck *et al*., 2013; Heagle, 1989; Heck, 1989). The total possible benefit of O_3_-resistant cultivar selection would almost certainly be larger than estimated here given the wide range of breeding materials available.

**Fig. 1:**
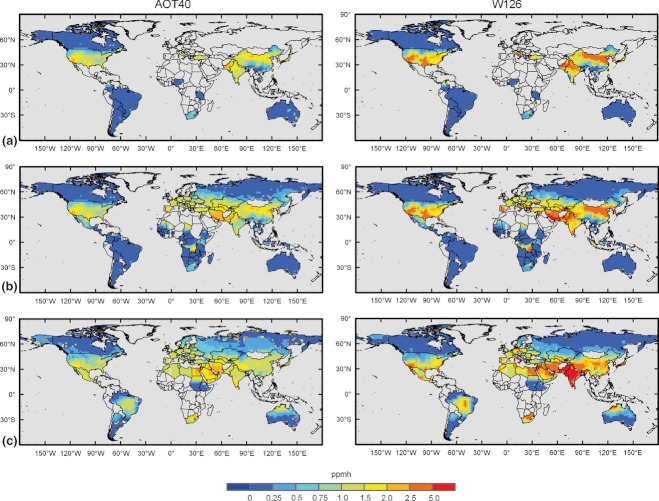
Global distribution of the reduction in year 2030 O_3_ exposure resulting from methane mitigation (CLE − CH_4_-red) according to AOT40 (left) and W126 (right) of (a) soybean, (b) maize, and (c) wheat during their respective growing seasons in each country (where crop calendar data are available). Minor producing nations not included in this analysis (where growing season data were unavailable) together account for <5% of global production of each crop (gray nations).

Using O_3_ exposure values in every grid cell for each metric and CR relationship (Fig. 1; Table S1), we calculate the relative yield (RY) of soybean, maize, and wheat and subtract this value from unity (representing a theoretical yield without O_3_ damage) to calculate relative yield loss (RYL). We then use satellite-based crop distribution maps (Monfreda *et al*., 2008; Ramankutty *et al*., 2008) (see SI; Fig. S2), which contain mean CP data per grid cell over the period 1997–2003, to convert grid cell RYL (%) into CPL (Mt) according to:


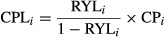


To find the gain in CP, we sum total CPL by country and calculate the difference in CPL between two scenarios in 2030, depending on the strategy examined: mitigation only, adaptation only, or both mitigation and adaptation, corresponding to CLE_med_ − CH_4_-red_med_, CLE_med_ − CLE_min_, and CLE_med_ − CH_4_-red_min_, respectively. We then multiply CP for each crop by national producer prices from the FAOSTAT database (FAO, 2008) to determine year 2030 total economic losses by country and globally. This simple revenue approach has been found to produce economic damage estimates within 20% of those based on a general equilibrium model accounting for factor feedbacks between crop yields, production, and commodity prices (Westenbarger & Frisvold, 1995).

For the mitigation-only scenario, we additionally provide a first-order estimate of the economic value of CP improvements from 2006 to 2030, as methane reductions would decrease ozone gradually over the period of mitigation. Following previous work, we use the annual average change in global surface O_3_ to scale year 2030 monetized benefits for agriculture over the 25-year period of CH_4_ (and O_3_) reductions (Fig. S3) (West & Fiore, 2005; West *et al*., 2006). We assume that agricultural benefits are linearly related to O_3_ reductions – a realistic assumption for AOT40 given its linear CR relationship, and for W126 within the range of O_3_ exposure values that generate the greatest agricultural losses (∼15–60 ppmh, Fig. S1). Furthermore, the spatial pattern of O_3_ reductions has been shown to be independent of the magnitude of CH_4_ emission changes over the simulated period (Fiore *et al*., 2008). We then calculate the present value of benefits from 2006 to 2030 using a 5% yr^−1^ discount rate, and amortize this sum to derive an estimate of constant annual benefits (with the same present value at the given discount rate) over the CH_4_ reduction period (West & Fiore, 2005; West *et al*., 2006).

## Results

### Reducing crop exposure to surface ozone with methane mitigation

We calculate the difference (CLE − CH_4_-red) in year 2030 crop exposure to O_3_ using two metrics (AOT40 and W126) as defined in Section Reductions in surface ozone exposure due to methane mitigation. Global average AOT40 and W126 over land, and CP-weighted average AOT40 and W126, during crop growing seasons are listed in Table 1 for year 2005 (i.e., before methane reductions start) and 2030 for the CLE and CH_4_-red scenarios (see SI for definitions).

**Table 1:** Global land-based average and crop production-weighted AOT40 and W126 in 2005 and 2030 under the CLE and CH_4_-red scenarios for each crop growing season, and percent change in O_3_ exposure due to CH_4_ mitigation in 2030 (relative to CLE). AOT40 and W126 values were calculated only for nations where growing season data were available, accounting for >95% of global production of each crop

Crop	AOT40 (ppmh)				W126 (ppmh)			
	
	2005	2030			2005	2030		
	
		CLE	CH_4_-red	%ΔO_3_		CLE	CH_4_-red	%ΔO_3_
Global average								
Soybean	4.4	5.8	5.3	7.9	4.8	6.9	6.2	9.7
Maize	4.2	5.3	4.8	8.9	4.4	6.0	5.4	10.6
Wheat	6.7	7.8	7.1	9.1	6.9	8.8	7.8	11.9
Production weighted								
Soybean	10.1	12.0	11.3	5.3	12.5	15.7	14.7	6.5
Maize	12.8	15.7	14.7	6.3	15.0	19.5	17.9	8.1
Wheat	10.0	14.0	12.9	7.6	10.1	18.3	15.2	17.1

For all three crops, simulated global average land-based AOT40 is higher than the European standard for the protection of agriculture in 2005 (3 ppmh, which is associated with a 5% reduction in crop yields) (LRTAP Convention, 2010). AOT40 is projected to be significantly higher for both 2030 scenarios, with production-weighted values of 10–12.8 ppmh in 2005 rising to 11.3–15.7 ppmh in 2030. Global land-based average W126 in 2005 is below the (recently withdrawn) proposed secondary O_3_ standard range in the United States (7–15 ppmh) (US Environmental Protection Agency, 2010) for all crops. Global average soybean- and maize-season W126 is also below the proposed standard in 2030 CLE, but wheat-season W126 is within the range. However, production-weighted W126 values are much higher, with W126 in 2030 CLE projected to rise well above the proposed secondary standard range (15.7–19.5 ppmh).

The spatial pattern of surface O_3_ exposure changes due to methane mitigation (CLE − CH_4_-red), as calculated by AOT40 and W126, is similar to the annual average tropospheric O_3_ change (Fiore *et al*., 2008), with the greatest reduction in O_3_ generally occurring from 0 to 30°N plus the southern Mediterranean (Fig. 1). The response of surface O_3_ to CH_4_ is primarily determined by the distribution of OH and NO_x_, and is strongest where surface air mixes frequently with the free troposphere and where the local O_3_ formation regime is NO_x_ saturated (Fiore *et al*., 2002, 2008; West & Fiore, 2005; West *et al*., 2006). Methane controls reduce global year 2030 O_3_ exposure by the greatest amount during the wheat growing season (9.1–11.9%, depending on the metric) due to the coincidence of this crop's growing regions with locations where the O_3_ response to CH_4_ controls is greatest (particularly India, Pakistan, Turkey, eastern China, and parts of the United States) (see Fiore *et al*. (2008), Fig. 1 and Fig. S2). The especially strong response of O_3_ during the wheat growing season in South Asia dominates the global CP improvements due to CH_4_ mitigation calculated in this analysis (see Section Year 2030 CP gains due to methane mitigation). Maize-season exposure is reduced by 8.9–10.6% and soybean exposure is reduced by 7.9–9.7% under the CH_4_-red scenario. The largest decrease in soybean exposure occurs in the United States, China, India, and Pakistan (reductions up to ∼2.9 and 5.2 ppmh with AOT40 and W126, respectively) (Fig. 1). Maize exposure is reduced substantially in the same countries plus Turkey, the southern Mediterranean, and parts of the Democratic Republic of Congo (up to ∼2.9 and ∼6 ppmh).

### Year 2030 CP gains due to methane mitigation

**Fig. 2:**
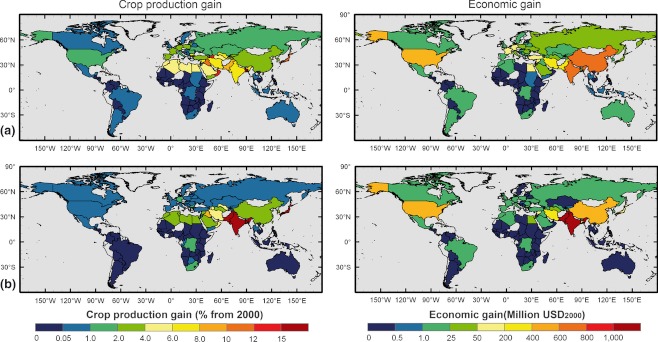
Total (soybean, maize, and wheat) year 2030 crop production (CP) gain in each nation due to CH_4_ mitigation as a percent increase from year 2000 production (left panels), and the estimated economic value (EV) of CP gains (right panels) according to (a) AOT40 and (b) W126. CP improvements represent the combination of estimated changes in O_3_ concentrations during specific crop growing seasons in regions where crops are grown, and the quantity of each crop produced in each nation. EV values also reflect national producer prices in addition to these factors.

We find that total (soybean, maize, and wheat) year 2030 CPL is projected to range from 224 to 243 Mt and 122 to 220 Mt under the CLE and CH_4_-red scenarios, respectively, depending on the metric used (Table 2). CPL is dominated by wheat in both scenarios, accounting for 77–85% of global losses of all three crops (Table 3). The controls on anthropogenic methane examined here would lead to a substantial reduction in CPL (i.e., CP gains) of 23–102 Mt (Table 2), with over 85% of the CP improvements due to wheat yield increases. Specifically, we project relatively small gains in soybean and maize production in 2030 (∼2–3 Mt each, an increase of ∼1% from year 2000 values), but much larger improvements for wheat (19–97 Mt, the equivalent of a 3.7–19% increase in year 2000 production) (Table 3). The methane controls in the CH_4_-red scenario could increase the combined year 2030 global production of soybean, maize, and wheat by 2.0–8.3% relative to 2000 values, worth $3.5–15 billion (all economic benefits are in USD_2000_). These CP gains due to CH_4_ mitigation represent the prevention of 10-45% of the O_3_-induced CPL that are otherwise projected to occur in 2030 CLE (Tables 2 and 4). CP improvements due to CH_4_ mitigation represent a substantial increase from year 2000 production in many regions of the world, particularly South Asia and parts of the Middle East (Fig. 2) where the O_3_ response to CH_4_ reductions is greatest (Fiore *et al*., 2008, 2009).

**Table 2:** Regionally aggregated combined soybean, maize, and wheat crop production loss (CPL, Mt) and its economic value (EV, billion USD_2000_) in 2030 under the CLE and CH_4_-red scenarios for each O_3_ exposure metric and concentration : response (CR) relationship examined here. The change in crop production (CP) and EV is shown, defined for AOT40 mean and W126 median as the difference between CLE and CH_4_-red CPL, and for W126 minimum (for both CLE and CH_4_-red) as the difference relative to the W126 median-derived CPL estimates in CLE. These scenarios are representative of a policy of methane mitigation, adaptation, and mitigation plus adaptation, respectively. For context, the change in CP is additionally presented as a percent of year 2000 crop production in each region. Note that this calculation is based on the increase (in Mt) from 2000 production values (i.e., representing a percent increase in production rather than relative yield). Regional definitions are available in Fig. S8

Region	Metric/CR relationship	2030 Crop production loss (Mt)				Economic value (billion USD_2000_)		
	
		CPL_CLE_	CPL_CH4-red_	ΔCP	%ΔCP (relative to 2000)	CLE	CH_4_-red	ΔEV
N. America	AOT40 – mean	52.5	48.4	4.1	1.0	6.4	6.0	0.5
	W126 – median	29.9	26.2	3.7	0.9	3.7	3.3	0.4
	W126 – minimum	10.2	—	19.8	4.7	1.1	—	2.6
	W126 – minimum	—	8.8	21.2	5.0	—	1.0	2.7
S. America	AOT40 – mean	1.8	1.6	0.2	0.1	0.22	0.19	0.03
	W126 – median	0.3	0.3	0.1	<0.1	0.04	0.04	0.01
	W126 – minimum	0.1	—	0.2	0.1	0.02	—	0.03
	W126 – minimum	—	0.1	0.2	0.2	—	0.02	0.03
Europe	AOT40 – mean	24.8	21.7	3.0	2.5	2.7	2.4	0.3
	W126 – median	2.3	1.8	0.5	0.4	0.3	0.3	0.0
	W126 – minimum	1.7	—	0.6	0.5	0.2	—	0.1
	W126 – minimum	—	1.2	1.1	0.9	—	0.1	0.2
Former Soviet Union	AOT40 – mean	10.5	9.21	1.3	1.2	1.3	1.1	0.2
	W126 – median	1.2	0.7	0.6	0.5	0.2	0.1	0.1
	W126 – minimum	0.9	—	0.3	0.3	0.1	—	0.1
	W126 – minimum	—	0.7	0.6	0.5	—	0.1	0.1
E. Asia	AOT40 – mean	48.9	43.6	5.3	2.4	6.6	5.9	0.7
	W126 – median	19.6	15.0	4.6	2.0	2.8	2.2	0.6
	W126 – minimum	10.1	—	9.5	4.2	1.4	—	1.5
	W126 – minimum	—	7.8	11.7	5.2	—	1.1	1.8
S. Asia	AOT40 – mean	88.5	81.6	7.0	5.9	13.3	12.2	1.1
	W126 – median	167	75.9	91.0	77.1	25.0	11.4	13.7
	W126 – minimum	55.8	—	111	94.2	8.4	—	16.7
	W126 – minimum	—	31.8	135	115	—	4.76	20.3
Africa & Middle East	AOT40 – mean	15.8	13.7	2.2	3.1	5.4	4.7	0.7
	W126 – median	3.9	2.6	1.3	1.9	1.4	0.9	0.5
	W126 – minimum	2.2	—	1.7	2.4	0.8	—	0.6
	W126 – minimum	—	1.6	2.3	3.3	—	0.6	0.8
Australia & Pacific	AOT40 – mean	0.4	0.3	0.1	0.2	0.39	0.32	0.1
	W126 – median	0.02	0.01	0.01	<0.1	<0.01	<0.01	<0.01
	W126 – minimum	0.01	—	0.02	<0.1	<0.01	—	<0.01
	W126 – minimum	—	0.0	0.02	0.1	—	<0.01	<0.01
World	AOT40 – mean	243	220	23.0	2.0	35.9	32.5	3.5
	W126 – median	224	122	102	8.3	33.5	18.2	15.3
	W126 – minimum	81.0	—	143	11.7	12.0	—	21.5
	W126 – minimum	—	52.0	172	14.1	—	7.7	25.8

**Table 3:** Global year 2030 soybean, maize, and wheat crop production loss (CPL, Mt) according to each O_3_ exposure metric and corresponding concentration : response (CR) relationship (i.e., median vs. minimum sensitivity) examined here for the CLE and CH_4_-red scenarios

	CPL (Mt)-AOT40		CPL (Mt)-W126			
	
Crop	CLE_med_	CH_4_-red_med_	CLE_med_	CH_4_-red_med_	CLE_min_	CH_4_-red_min_
Soybean	27.9	25.9	16.7	15.2	4.00	3.71
Maize	22.8	20.7	16.1	13.2	7.18	5.63
Wheat	192	173	191	94.0	69.8	42.7

**Table 4:** Summary of global crop production benefits (and their economic value) in 2030 due to different policy choices: methane mitigation only, adaptation only (choice of O_3_-resistant cultivars), and both mitigation and adaptation. Crop production (CP) increases in Mt are also represented as a percent reduction in O_3_-induced crop production loss (CPL) relative to CLE_med_ in 2030, and as a percent increase from year 2000 crop production

Policy choice	Scenarios	Metric	ΔCP (Mt)	%ΔCPL (from CLE_med_)	%ΔCP (from 2000)	Economic benefit (billion USD_2000_)
Mitigation only	CLE_med_ − CH_4_-red_med_	AOT40	23	10	2.0	3.5
Mitigation only	CLE_med_ − CH_4_-red_med_	W126	102	45.4	8.3	15
Adaptation only	CLE_med_ − CLE_min_	W126	143	63.9	11.7	22
Mitigation and adaptation	CLE_med_ − CH_4_-red_min_	W126	172	76.8	14.1	26

Economic benefits are concentrated in regions of major production, primarily the United States, China, and India. South Asia is projected to experience the greatest economic benefit, driven by improvements in yields of O_3_-sensitive wheat: ∼7–91 Mt worth ∼$1.1–14 billion (Table 2). We note, however, that because 2030 CLE O_3_ exposure in this region is based on significant projected growth of O_3_ precursor emissions from 2000 to 2030 (e.g., NO_x_ ∼x2) (Dentener *et al*., 2005), recently introduced and future emission control legislation may lead to lower O_3_ levels than predicted here (Van Dingenen *et al*., 2009). The estimated benefit of CH_4_ mitigation may therefore be overly optimistic in this region. East Asia is estimated to experience significant gains (4.6–5.3 Mt) from CH_4_ mitigation due primarily to soybean production improvements worth $600–700 million in 2030. North American CP gains are driven primarily by O_3_ reductions that occur during the soybean and maize growing season (Fig. 1). These gains are expected to increase CP by 3.7–4.1 Mt with a value worth over $400–500 million in the year 2030 (Table 2).

Previous estimates of the economic benefits of CH_4_ mitigation have accounted for the value of recovered methane and the averted adverse human health effects of O_3_ reductions (West & Fiore, 2005; West *et al*., 2006, 2012). Here, we provide an estimate of the agricultural benefits alone, over the period of CH_4_ control (2006–2030) (see Section Crop production and economic gains). We find the present value of agricultural gains through 2030 to be $17–75 billion USD_2000_ (amortized to $1.2–5.3 billion yr^−1^), substantially increasing the cost effectiveness of CH_4_ mitigation. Global marginal benefits for agriculture in 2030 (estimated here to a first order as the global year 2030 economic benefit (Table 2) divided by the methane reductions in 2030 (125 Mt) and discounted at 5% yr^−1^) are calculated to be $8–36 per ton CH_4_ reduced (depending on the metric), improving the cost effectiveness of the methane reduction policy by 5–22% (based on the mitigation cost estimate of West *et al*., 2012).

### Explaining differences in estimates of CP improvement from methane mitigation

The large discrepancy between global AOT40- and W126-derived estimates of potential CP improvements is driven by the different projected wheat CPL in the CH_4_-red scenario, the majority of which occurs in the Indian subcontinent as previously highlighted (Tables 2 and 3). This discrepancy partly results from differences in the calculated O_3_ exposure reduction estimated by each metric. W126 accounts for hourly O_3_ across the spectrum of concentrations rather than solely O_3_ levels above 40 ppbv. As methane reductions decrease O_3_ by a similar amount across the whole distribution of O_3_ levels (Fiore *et al*., 2002; West & Fiore, 2005), concentrations below 40 ppbv are also affected. More importantly, however, are the different weights assigned to hourly O_3_ concentrations that are incorporated into the cumulative metric calculations. Figure S4 illustrates the functions used to weigh hourly O_3_ concentrations for both metrics: the AOT40 weighting function is steepest just above 40 ppbv and progressively flattens, whereas the W126 function assigns significantly greater weight to O_3_ concentrations above the inflection point of the weighting curve (∼62 ppbv). This in turn may generate substantially different calculated changes in O_3_ exposure due to CH_4_ mitigation as derived by each metric, depending on local O_3_ concentrations, as weighted hourly O_3_ concentrations are accumulated over the growing season. For example, in the Indian subcontinent, O_3_ exposure defined by W126 is reduced by 7.6 ppmh (∼18%; Fig. 1) under the CH_4_-red scenario, but defined by AOT40 is only reduced by 1.3 ppmh (∼5%). The greater change in O_3_ exposure projected by the W126 metric, combined with the steep slope of the W126 CR relationship for wheat at high levels of O_3_ exposure (Fig. S1) and the large amount of wheat grown in the Indian subcontinent (Fig. S2), leads to substantially greater projected CP improvements in India when calculated by W126 rather than AOT40.

### Year 2030 CP gains due to cultivar selection

We use the W126 metric to quantify the potential year 2030 benefits of selecting soybean, maize, and wheat cultivars with the greatest demonstrated tolerance to ozone (i.e., minimum sensitivity varieties, CLE_min_) relative to baseline crop sensitivity to O_3_ (i.e., median or mean crop sensitivity, CLE_med_), as described in Section Crop production and economic gains. We follow the methods discussed in Section crop production and economic gains to calculate CP gains, here defined as the difference between CPL in 2030 CLE derived from the two different parameterizations of the W126 CR function (CLE_med_ − CLE_min_).

**Fig. 3:**
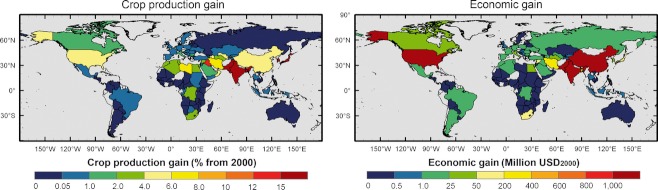
Total (soybean, maize, and wheat) year 2030 crop production (CP) gain in each nation due to cultivating O_3_ tolerant crops (CLE_min_) relative to the median sensitivity of cultivars analyzed in US field studies (CLE_med_), represented as a percent increase from year 2000 production (left). The estimated economic value (EV) of CP gains is also shown (right).

We find that total (soybean, maize, and wheat) year 2030 CPL to be 81 Mt for CLE_min_, an increase in production of 143 Mt from CLE_med_. This is the equivalent of an ∼12% improvement in year 2000 production and is projected to be worth ∼$22 billion (Table 2). CP gains are once again highest for wheat (Table 3): 122 Mt relative to CLE_med_ (an increase of ∼24% from year 2000 production), representing the prevention of ∼64% of the CPL otherwise projected to occur in 2030 (Table 3, columns 4 and 6). However, we project substantially greater increases in soybean and maize production when the O_3_-resistant cultivar is chosen than with the policy of CH_4_ control. By choosing a minimally sensitive cultivar, global soybean and maize CP improves relative to 2000 by 8.0% and 1.6%, respectively, with total increases in these crops (∼22 Mt) representing a 55–76% reduction in the losses expected to occur with cultivars of median sensitivity in 2030 (Table 3, columns 4 and 6). For this reason, the adaptation strategy provides significantly greater benefits than methane mitigation in regions where soybean and maize are the primary sources of CPL (e.g., North America and East Asia) (Table 2; Fig. 3). CP gains are expected to be highest in the Indian subcontinent where the rise in O_3_ is projected to be greatest under CLE from 2005 to 2030: planting the more O_3_-resistant crop cultivars (particularly for wheat) would increase total CP by 111 Mt from CLE_med_, the equivalent of >90% of regional production in 2000 (Table 2). We find that India and Pakistan would accrue the greatest economic benefit from increased selection for O_3_ tolerance (∼$16 billion combined and ∼74% of global economic benefits), followed by the United States ($2.5 billion) and China ($1.2 billion) (Fig. 3).

### Year 2030 CP gains due to methane mitigation and cultivar selection

We follow the same approach outlined in Section Materials and methods to explore the benefits to agriculture of both mitigation and adaptation policies in 2030; in this case we compare W126 minimum sensitivity cultivars and the CH_4_-red scenario (CH_4_-red_min_) with CLE_med_. Table 4 summarizes global CP and economic benefits for each policy scenario we explore. We find that total (soybean, maize, and wheat) year 2030 CPL is projected to be 52 Mt for CH_4_-red_min_, representing an increase in global production of 172 Mt from CLE_med_. This is the equivalent of a 14% increase in year 2000 production and is projected to be worth ∼$26 billion (Table 2). Employing both mitigation and adaptation strategies would reduce ∼77% of the O_3_-induced CPL expected to otherwise occur in 2030 (relative to CLE_med_), compared with a reduction in CPL of ∼45% and 64% with CH_4_ mitigation and adaptation alone, respectively (Table 4). Wheat gains account for the majority of the total CP and economic improvements when both strategies are simultaneously applied (Table 3). For this reason, South Asia receives the greatest additional benefit from combining both mitigation and adaptation strategies (Table 2; Fig. S5). The added agricultural production arising from employing the adaptation strategy in addition to CH_4_ mitigation includes ∼12, 8, and 51 Mt of soybean, maize, and wheat (Table 3, columns 5 and 7) worth ∼$10.5 billion globally in 2030 (Table 2, column 8). Increased soybean, maize, and wheat production due to CH_4_ abatement in addition to cultivar selection alone is estimated to be ∼0.3, 1.5, and 27 Mt, respectively, in 2030 (Table 3, columns 6 and 7) worth $4.3 billion globally (Table 2, columns 7 and 8). The benefits to agriculture of combining both strategies are less than fully additive because the benefits of adaptation are highest at elevated levels of O_3_ exposure where the greatest damages to crops occur (evident from the shape of the W126 CR functions, Fig. S1).

## Discussion

### Major sources of uncertainty

An important source of uncertainty in this study is the use of simulated hourly O_3_ concentrations by a global CTM to predict future O_3_ exposure. O_3_ concentrations simulated by MOZART-2 and used in this analysis have been extensively evaluated (Fiore *et al*., 2008, 2009), with additional evaluation shown in Table S2. MOZART-2 performs well overall with few exceptions – notably a bias of >10 ppb in summer over the eastern United States and Japan (Fiore *et al*., 2008, 2009), a common bias in global models (Fiore *et al*., 2009; Reidmiller *et al*., 2009). Of particular importance to our results is model performance in South Asia, and O_3_ is well simulated in rural northern India where most wheat is grown (Fig. S2, Table S2), although the model overestimates O_3_ in southern India (mean bias of ∼+10 ppbv) based on limited observations from the years 2002–2005. The paucity of representative O_3_ observations in India with which to evaluate model performance, combined with the importance of this region in driving global results, introduces uncertainty into the magnitude of the estimated benefits to agriculture derived here. See the SI for further discussion.

Another major source of uncertainty in this study is the projected emissions of future O_3_ precursors. The CLE scenario includes emission control legislation enacted through 2001, but more recently introduced policies are unaccounted for and may therefore lead to lower O_3_ levels than simulated here – and correspondingly to reduced benefits for agriculture as a consequence of mitigating O_3_ via CH_4_ abatement. However, the CLE scenario assumes perfect compliance with O_3_ regulations, a highly optimistic assumption about actual policy implementation and enforcement (particularly in rapidly industrializing nations), which may counterbalance this effect.

Our global application of CR relationships derived from field studies in the United States and Europe in the 1980s/1990s (due to the lack of similar large-scale studies elsewhere) is an additional significant source of uncertainty. Crop cultivars currently grown may have different sensitivities to O_3_ than those derived previously. However, recent field research indicates that current crop sensitivity is at least as great as that found in earlier studies in the United States (Long *et al*., 2005; Morgan *et al*., 2006), and that CR functions derived in North America and Europe in fact underestimate the effects of O_3_ on crop yields in Asia (Emberson *et al*., 2009; Zhu *et al*., 2011; Wang *et al*., 2012). In the SI, we use recently derived CR functions for Chinese cultivars of wheat to estimate CPL under the CLE and CH_4_-red scenarios. We find that wheat in China is projected to suffer O_3_-induced CPL that are ∼50% greater than predicted according to Western CR functions, with the CP improvements due to methane mitigation estimated to be ∼70% higher in China and across East Asia (increasing from 4.3 to 7.4 Mt, Table S3). Estimated benefits to agriculture are therefore particularly uncertain in South and East Asia due to uncertainties about relative crop sensitivity in addition to model performance. Errors in estimated O_3_-induced wheat loss in South Asia could significantly affect the total calculated CP improvements derived here given the importance of South Asian wheat to global results.

Our calculation of monetized benefits for agriculture due to CH_4_ reductions and O_3_-resistant crop cultivar selection neglects future changes in commodity prices and in agricultural production. Because both will likely increase substantially over the next few decades in response to a growing population, shifting diets, and the increasing use of biofuel (Food & Agriculture Organization of the United Nations, 2006), this simplification likely leads to an underestimate of O_3_-induced crop losses and therefore of the total agricultural and economic benefits of CH_4_ mitigation and cultivar selection. Furthermore, as our CH_4_-red simulation is not at steady state, O_3_ reductions due to CH_4_ controls would continue beyond 2030 – these benefits are not included in our analysis.

Changes in regional climate over the next few decades may affect O_3_ concentrations and distributions (which are not accounted for by MOZART-2), but such changes are expected to be of second order compared to those driven by anthropogenic emissions of CH_4_ and other ozone precursors in most land regions (Dentener *et al*., 2006). However, climate change may have a greater impact on O_3_-induced crop yield reductions through its affect on the stomatal conductance of O_3_. Increased temperatures and atmospheric CO_2_ concentrations and decreased humidity and soil water content in some regions may reduce stomatal openings and therefore the amount of O_3_ that enters into plants and resulting damage (Mauzerall & Wang, 2001; Fuhrer, 2009). Flux-based metrics have been shown to more accurately predict the yield response of some crops to present-day O_3_ concentrations and have been used to evaluate possible O_3_ damages in a future climate (Pleijel *et al*., 2004; Mills *et al*., 2011a However, unfortunately data do not yet exist to apply flux-based approaches globally.

An important limitation of the exposure metrics used here is that they may overestimate O_3_-induced agricultural damages in nonirrigated, drier regions of the world (where water stress may induce stomatal closure), or those regions predicted to become drier in the next few decades (assuming no additional irrigation). The EPA estimates that O_3_ exposure values (as defined by W126) would need to more than double to induce the same RYL in drought vs. well-watered conditions (Fig. S6). Water stress therefore provides an important measure of protection against O_3_ that is not accounted for here for the ∼60% of cereal production that is grown on rainfed cropland. In the SI, we estimate, to a first order, that not accounting for water stress over rainfed regions may lead to benefits that are overestimated by approximately 38% (Table S4 and Supporting Text).

However, CP improvements predicted for wheat in South Asia contribute most significantly to our calculated global benefits due to reducing O_3_ damages to crops. Wheat in this region is heavily irrigated – estimated at over 91% in 2011 (Fig. S7) (Singh, 2012). Irrigation in general is more widely used in many developing countries where a substantial portion of CP gains is predicted: for example, 70% of Chinese grains are grown on irrigated land, 50% in India, and 15% in the United States (Brown *et al*., 2002). CPL, and gains due to O_3_ abatement, may be overestimated in North America, north/central Europe, and Latin America where rainfed crops dominate (Fig. S7). However, the coincidence of highly productive regions in Asia (particularly for wheat in India and China) with regions of substantial irrigation (Figs. S2 and S7) suggests that our results on a global level may not be significantly biased by the use of exposure-based metrics. See the SI for additional discussion.

Although water stress may thus protect against O_3_ in some regions, O_3_ exposure may leave crops more susceptible to other biotic and abiotic stressors. Unfortunately, the overall impact of and interactions between various environmental stressors (e.g., heat waves, drought, pests, etc.) is poorly understood. Additional field studies (including OTC as well as fully open-air field experiments) using a variety of cultivars grown around the world (particularly in Asia) under different field conditions would reduce uncertainties about relative crop sensitivity to O_3_ in different regions, as well as improve our understanding of the effect of multiple environmental stressors and future climate changes on O_3_ sensitivity. The continued development of flux-based models for estimating O_3_ impacts in important agricultural regions across the globe (e.g., the United States, India, and China) will additionally allow for more accurate assessments of O_3_ risk to regional and global CP.

### Policy implications

In stark contrast to the gains in crop productivity made during the Green Revolution, studies suggest that growth in crop yields in many parts of the world have recently been in decline (Tilman *et al*., 2002; World Bank, 2007; Dasgupta & Sirohi, 2010). Increasing evidence points to elevated levels of O_3_ as an additional and extremely important (yet overlooked) factor in this deceleration of crop yield growth (Wang & Mauzerall, 2004; Van Dingenen *et al*., 2009; Fishman *et al*., 2010; Avnery *et al*., 2011a Zhu *et al*., 2011). Our current study follows earlier work which quantified the present and potential future (year 2030) impact of surface O_3_ on the global yields of soybean, maize, and wheat given both upper- and lower-boundary projections of reactive O_3_ precursor emissions (Avnery *et al*., 2011a,b). The latter study (Avnery *et al*., 2011b found substantial future yield losses globally for these crops even under a scenario of stringent O_3_ control via traditional pollution mitigation measures (i.e., reductions in NO_x_, VOCs, and NMVOCs): 10–15% for soybean, 3–9% for maize, and 4–17% for wheat.

Given the potential for significant future O_3_-induced yield losses, in this study we present two additional strategies to reduce O_3_ damages to crops beyond targeting traditional O_3_ precursors (CH_4_ controls and selection for more O_3_-resistant crop cultivars, as well as their combination) – and thereby to increase future agricultural production without further harming the environment. We find that the anthropogenic methane reductions examined here could yield global CP gains for soybean, maize, and wheat of ∼2–8% in 2030 relative to year 2000 production, worth $3.5–15 billion in 2030 and $17–75 billion ($1.2–5.3 billion yr^−1^) from 2006 to 2030. We further find that choosing cultivars with high O_3_ resistance could increase year 2030 CP by ∼12% relative to year 2000, with an economic value of ∼$22 billion. Combining both CH_4_ mitigation and cultivar adaptation strategies could increase global CP by 14% from 2000, worth $26 billion worldwide (Table 4).

Although we find that the adaptation-only strategy may provide higher potential agricultural benefits than O_3_ abatement through methane control, we do not suggest that cultivar selection is superior or that it should be pursued in lieu of O_3_ mitigation. Ozone is detrimental to human health, and the modest CH_4_ controls examined here could prevent 411 000 premature mortalities via their surface ozone reductions through 2030 (West *et al*., 2012). In addition, the methane abatement policy examined here would have major benefits for climate change by offsetting positive net radiative forcing from CH_4_ and O_3_ projected to otherwise occur by 2030 (∼0.16 Wm^−2^) (Fiore *et al*., 2008). Moreover, CH_4_ controls and corresponding O_3_ reductions would increase the carbon storage potential of forests and other ecosystems that would arise from reduced O_3_ damages to vegetation (Felzer *et al*., 2005, 2007). These indirect effects may have a greater impact on climate than the direct radiative forcing of tropospheric O_3_ (Sitch *et al*., 2007).

In addition, with an atmospheric lifetime of ∼12 years, CH_4_ is considered a short-lived climate forcer (SLCF). Interest in reducing emissions of SLCFs (including methane, black carbon (BC), and many hydrofluorocarbons) has been growing as a strategy to reduce the rate of climate warming and the risk of abrupt climate change (Molina *et al*., 2009). A global effort to catalyze rapid reductions in these species has been initiated by the recently formed United Nations Environment Program (UNEP) Climate and Clean Air Coalition to Reduce Short-Lived Climate Pollutants (http://www.unep.org/ccac/). UNEP and the World Meteorological Organization (WMO) estimate that technically feasible reductions in CH_4_ and BC emissions (the latter of which, by targeting many of the same sources, would also reduce O_3_ precursors CO and NO_x_) could decrease warming in 2050 by 0.5 °C and reduce the likelihood of crossing the 2 °C temperature threshold considered ‘dangerous anthropogenic interference with the climate system’ (UNEP & WMO, 2011; Shindell *et al*., 2012). The authors calculate that these reductions in CH_4_ and BC would additionally prevent 0.7–4.7 million premature mortalities and increase global CP by 30–135 Mt in 2030, with the greatest health and agricultural benefits accruing to South and East Asia (led by India and China, as we find here). Although benefits estimated by Shindell *et al*. (2012) are slightly lower than presented in this study, CP gains were assessed using daytime average metrics of O_3_ exposure (M7/M12) that project substantially lower O_3_-induced yield losses for wheat than the cumulative AOT40 and W126 indices (Wang & Mauzerall, 2004; Van Dingenen *et al*., 2009; Avnery *et al*., 2011a which are considered more accurate predictors of crop yield response to O_3_ (Lefohn & Runeckles, 1988; Lesser *et al*., 1990).

Of the methane abatement measures examined by Shindell *et al*. (2012) (which represent a 38% reduction in reference scenario emissions by 2030), approximately one third target oil and gas production in North America, Europe, and parts of Asia; another one third address emissions from coal mining, especially in South and East Asia; and most of the remaining CH_4_ reductions are generated by improving agricultural and municipal waste management practices globally. About half of the identified emission controls could be implemented at a cost savings, with another third achieved at low-to-moderate cost. O_3_ mitigation via methane reductions described here and elsewhere (Fiore *et al*., 2008; Shindell *et al*., 2012) should therefore be considered an effective strategy for long-term international air quality management with major climate change, agricultural, and health cobenefits. Methane abatement would complement local policies to reduce conventional O_3_ precursors, in particular NO_x_ emissions, which could generate both local and global benefits to agriculture due to a decrease in transboundary O_3_ transport (Hollaway *et al*., 2012). Major agricultural producers of South Asia, East Asia, and North America (e.g., India, China, and the United States, Fig. 2) have a particular incentive to reduce O_3_ given the substantial projected O_3_-induced crop losses in these regions. Reducing O_3_ damages to crops may be an especially attractive food security strategy in India, a nation facing predicted population growth of almost 300 million people by 2030 (United Nations Population Division, 2010) while over 20% of its population was undernourished in 2005–2007 (Food & Agriculture Organization of the United Nations, 2009), and where arable land and water resources are becoming increasingly strained. Adaptive strategies such as cultivar selection should further supplement O_3_ mitigation to maximize global CP, particularly in regions where agriculture is vulnerable to rapidly rising O_3_ concentrations. Although considerable uncertainties remain, O_3_ mitigation and/or increasing ozone resistance among cultivated crop varieties thus provides important opportunities to significantly improve future CP without further environmental degradation.

## Supporting Information

**Figure S1.** Concentration : response (CR) functions for wheat used in this analysis for AOT40 (solid line), median sensitivity W126 (small dashes), and minimum sensitivity W126 (representing maximum O_3_ resistance) (large dashes). Equations for these functions are listed in Table S1.

**Figure S2.** Global distributions of soybean, maize, and wheat crop production in the year 2000.

**Figure S3.** Change in global annual surface O_3_ (solid) used to scale estimated agricultural benefits in 2030 over the 25-year period of CH_4_ reductions (dashed).

**Figure S4.** Weighting curves applied to hourly O_3_ concentrations used to calculate the cumulative AOT40 and W126 metrics.

**Figure S5.** Total (soybean, maize, and wheat) year 2030 crop production (CP) gains in each nation resulting from the combined benefits of CH_4_ mitigation and minimum O_3_-sensitivity cultivar choice (CLE_med_ − CH_4_-red_min_) relative to CLE_med._ Results are presented as a percent increase from year 2000 production (Monfreda *et al*., 2008; Ramankutty *et al*., 2008) (left). The estimated economic value (EV) of CP gains is also shown (right).

**Figure S6.** W126 CR functions (median response, or 50th percentile of all crops and cultivars) for well-watered and droughted conditions, based on pooling data from eight original National Crop Loss Assessment Network (NCLAN) field studies that paired droughted and well-watered conditions for the same genotype (Lee & Hogsett, 1996).

**Figure S7.** Global map of irrigated areas (percent of grid cell irrigated), based on data from Siebert *et al*. (2010).

**Figure S8.** Regional definitions used to calculate crop production losses in Table 2.

**Table S1.** Concentration : response equations used to calculate relative yield loss of soybean, maize, and wheat.

**Table S2**. Regionally averaged ratios of modeled:observed O_3_ exposure according to different metrics (depending on data availability) during the wheat growing season in each region.

**Table S3.** Wheat crop production losses (CPL) and crop production (CP) improvements in East Asia due to methane mitigation estimated according to an AOT40 concentration : response (CR) function derived from Chinese cultivars of wheat (Wang *et al*., 2012), compared with mean sensitivity wheat CR functions based on US and European field studies (Table S1).

**Table S4.** Calculated crop production loss (CPL) and crop production (CP) improvement due to CH_4_ mitigation according to all-crop, well-watered, and droughted median CR functions (Table S1) as applied to wheat. Calculations assume 100% of wheat is rainfed and therefore either fully droughted or well-watered; in actuality, only ∼60% of crops are rainfed.
