# Supplementary figures and images for: Increasing global agricultural production by reducing ozone damages via methane emission controls and ozone-resistant cultivar selection
Source: Glob Chang Biol. 2013 Feb 5;19(4):1285–99. doi: 10.1111/gcb.12118 (PMC3627305; doi:10.1111/gcb.12118)

# Wheat Sensitivity to Ozone

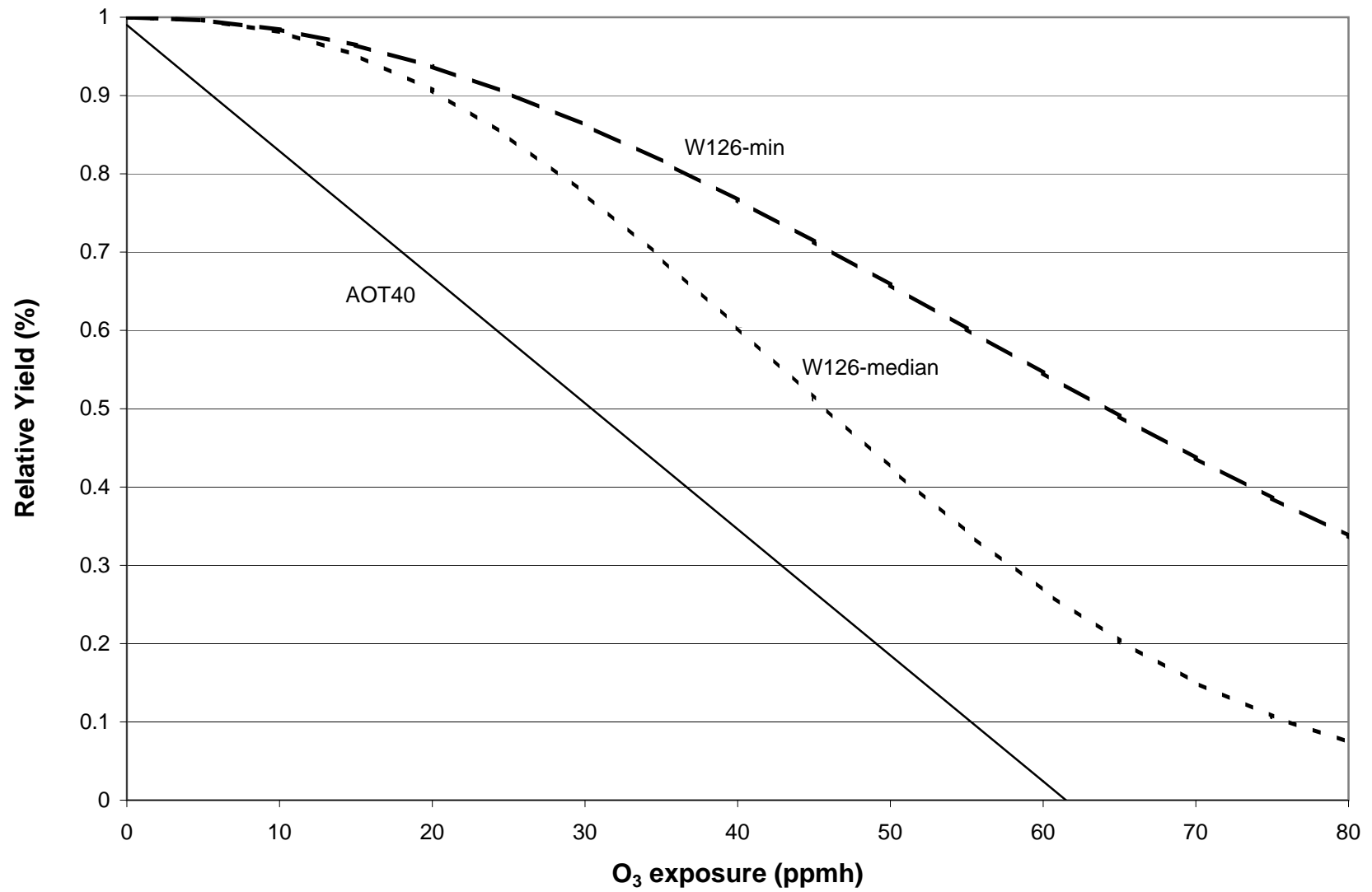

Supplement: Supplementary file 1 [file gcb0019-1285-SD1.pdf]

Soybean

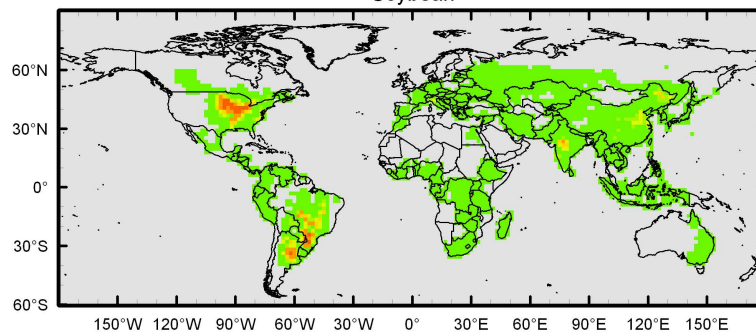

Maize

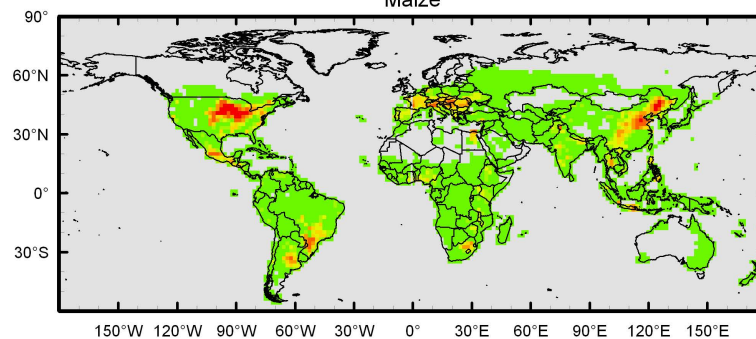

Wheat

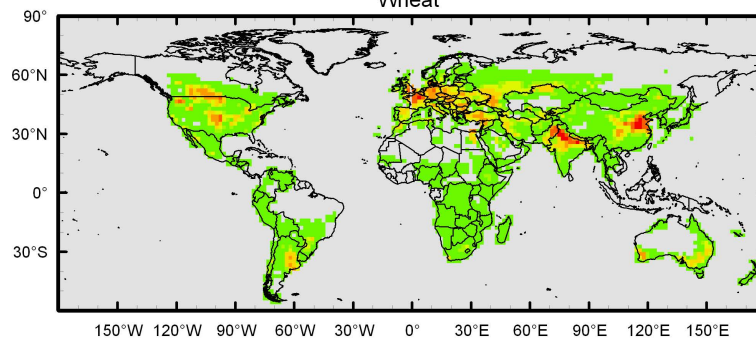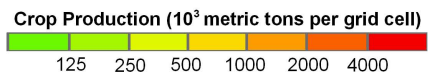

Supplement: Supplementary file 2 [file gcb0019-1285-SD2.pdf]

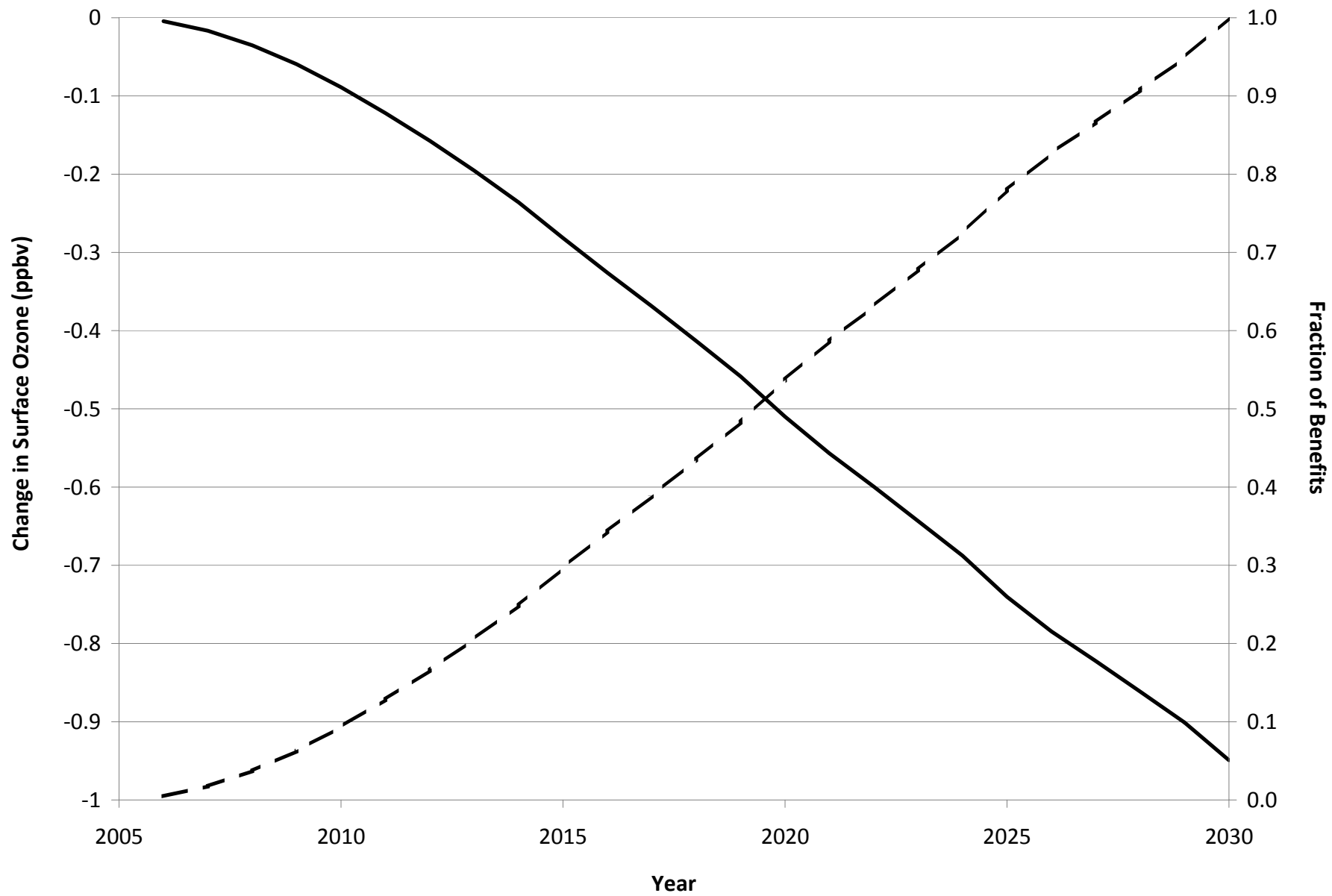

Supplement: Supplementary file 3 [file gcb0019-1285-SD3.pdf]

## AOT40 and W126 Weighting Functions

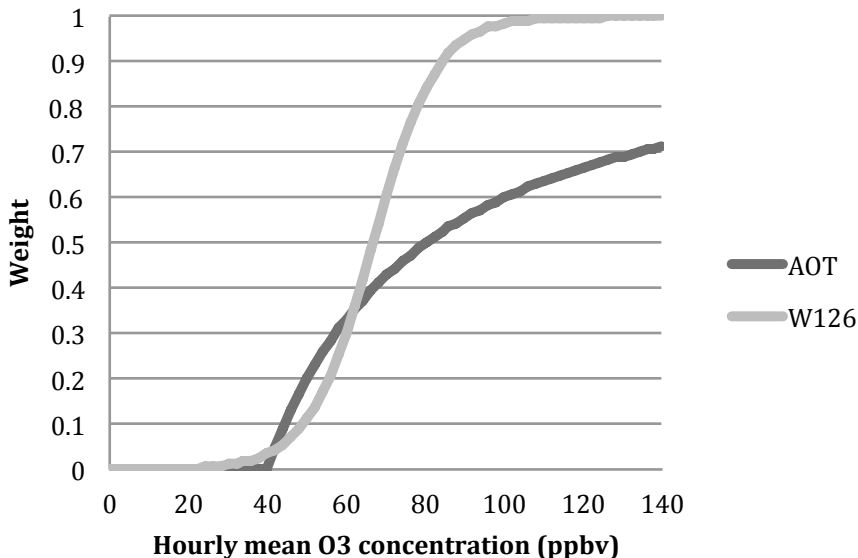

Supplement: Supplementary file 4 [file gcb0019-1285-SD4.pdf]

Crop Production Gain

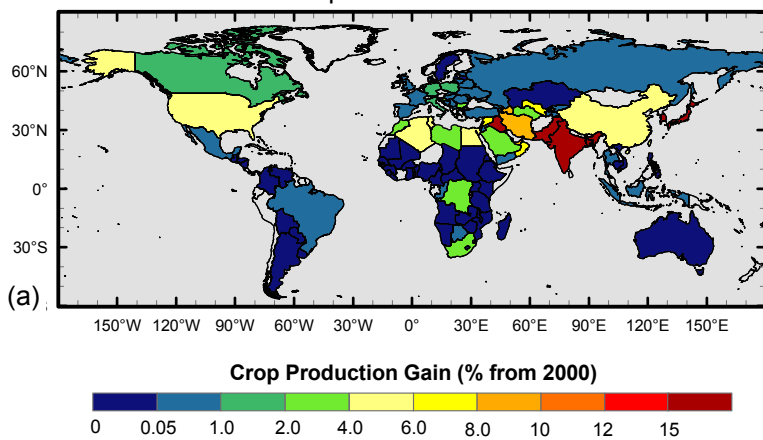

Economic Gain

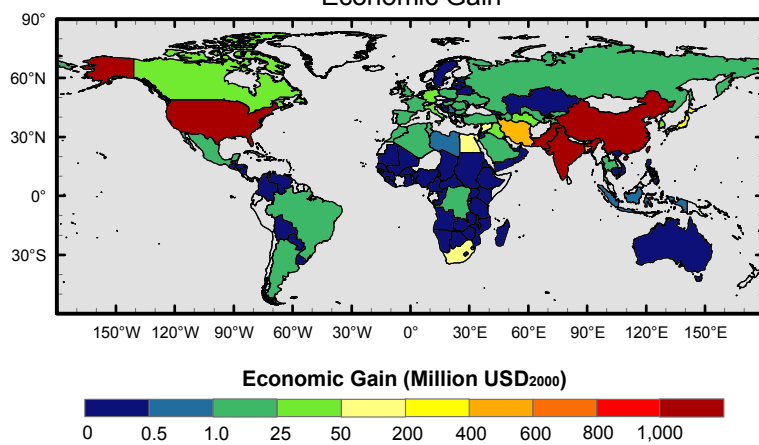

Supplement: Supplementary file 5 [file gcb0019-1285-SD5.pdf]

## All Crop Sensitivity to Ozone

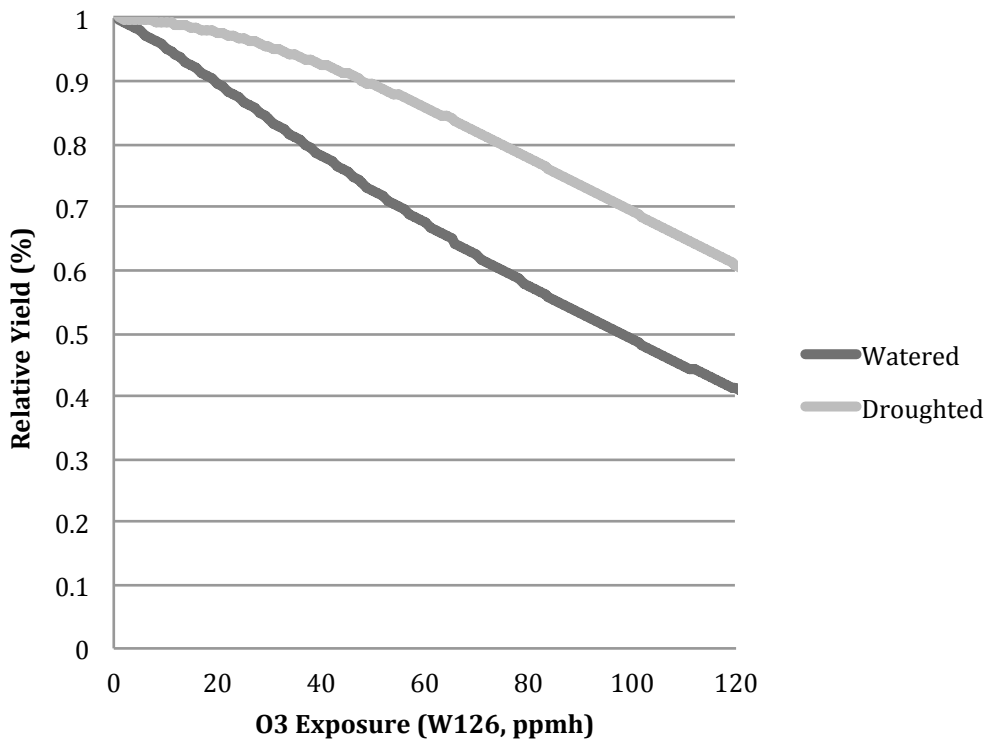

Supplement: Supplementary file 6 [file gcb0019-1285-SD6.pdf]

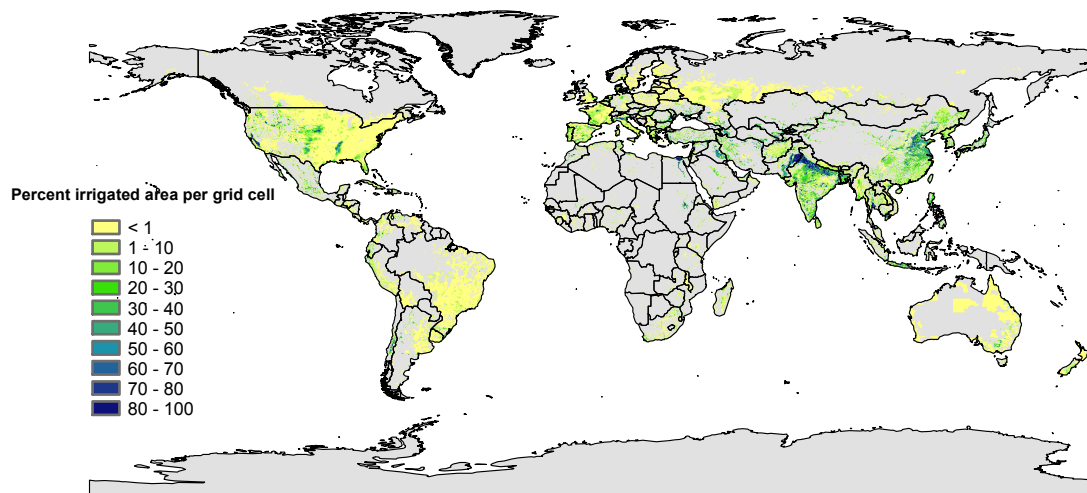

Supplement: Supplementary file 7 [file gcb0019-1285-SD7.pdf]

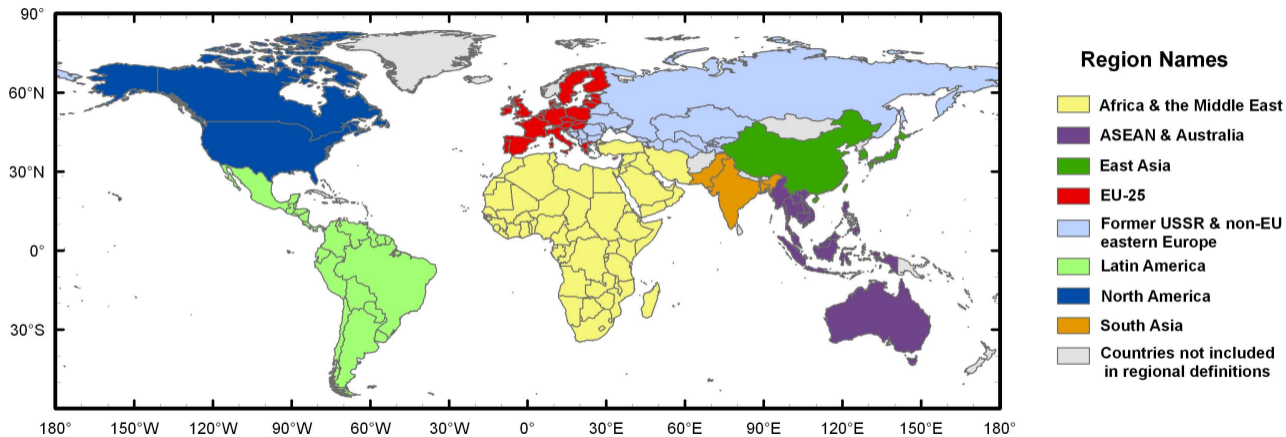

Supplement: Supplementary file 9 [file gcb0019-1285-SD8.pdf]
