# Supplementary material for: Increasing global agricultural production by reducing ozone damages via methane emission controls and ozone-resistant cultivar selection
Source: Glob Chang Biol. 2013 Feb 5;19(4):1285–99. doi: 10.1111/gcb.12118 (PMC3627305; doi:10.1111/gcb.12118)
Supplement: Supplementary file 10 [file gcb0019-1285-SD9.docx]

| **Crop** | **Concentration – Relative Yield Relationship** | **Relative Sensitivity** | **Reference** |
| --- | --- | --- | --- |
| Soybean | *RY* = -0.0116**AOT40*+1.02 | Average | Mills *et al*. (2007) |
|  | *RY* = exp[-(*W126*/110.2)^1.359^] | Median | Lee & Hogsett (1996) |
|  | *RY* = exp[-(*W126*/476.7)^1.113^] | Minimum | Lee & Hogsett (1996) |
| Corn | *RY* = -0.0036**AOT40*+1.02 | Average | Mills *et al*. (2007) |
|  | *RY* = exp[-(*W126*/97.9)^2.966^] | Median | Lee & Hogsett (1996) |
|  | *RY* = exp[-(*W126*/94.2)^4.167^] | Minimum | Lee & Hogsett (1996) |
| Wheat | *RY* = -0.0161**AOT40*+0.99 | Average | Mills *et al.* (2007) |
|  | *RY* = 1-.022795**AOT40* | Average – Chinese cultivar^a^ | Wang *et al*. (2012) |
|  | *RY* = exp[-(*W126*/53.4)^2.367^] | Median | Lee & Hogsett (1996) |
|  | *RY* = exp[*-*(*W126*/76.8)^2.031^] | Minimum | Lee & Hogsett (1996) |
| All-crop^b^ | *RY =* exp[-(*W126*/132.86)^1.170^] | Median – Well Watered | Lee & Hogsett (1996) |
|  | *RY* = exp[-(*W126*/179.84)^1.713^] | Median – Droughted | Lee & Hogsett (1996) |

^a^ Used to compare yield losses derived from U.S./European concentration:response (CR) functions with those calculated using a CR function derived from field studies in China.

^b^ Based on pooling crop response data from eight U.S. field studies that paired droughted and well-watered conditions for the same genotype.

**Table S1.**  Concentration:response equations used to calculate relative yield loss of soybean, maize, and wheat. RY = relative yield as compared to theoretical yield without O_3_-induced injury. Relative yield loss (RYL) is calculated as (1 – RY). See manuscript text for definitions of AOT40 and W126. For the AOT40 CR relationship, we use parameter values pooled from a variety of cultivars grown in the U.S. and Europe for each crop representing the best-fit linear response (Mills *et al*., 2007). For the W126 CR function, we use median parameter values (representative of the 50^th^ percentile crop response) pooled from U.S. cultivars (Lee & Hogsett, 1996).
