# Supplementary material for: Increasing global agricultural production by reducing ozone damages via methane emission controls and ozone-resistant cultivar selection
Source: Glob Chang Biol. 2013 Feb 5;19(4):1285–99. doi: 10.1111/gcb.12118 (PMC3627305; doi:10.1111/gcb.12118)
Supplement: Supplementary file 11 [file gcb0019-1285-SD10.docx]

| **Region** | **M12 (M24)** | **AOT40** | **W126** | **Minimum Lon, Lat** | **Maximum Lon, Lat** | **Number of Stations** | **Data Source** |
| --- | --- | --- | --- | --- | --- | --- | --- |
| U.S. Midwest | 1.11 | 1.60 | 1.63 | -95, 35 | -84, 50 | 128 | EPA Air Quality System (AQS), (http://www.epa.gov/ttn/airs/airsaqs/) |
| U.S. Northeast | 1.13 | 2.06 | 2.13 | -83, 35 | -65, 50 | 232 | AQS |
| U.S. Central/West | 1.14 | 1.24 | 1.17 | -155, 30 | -96, 50 | 246 | AQS |
| Central Mediterranean | 1.06 | 1.15 | 0.79 | -10, 40 | 35, 47 | 43 | European Monitoring and Evaluation Programme (EMEP) (http://www.nilu.no/projects/CCC/onlinedata/ozone/index.html) |
| Central Europe | 1.04 | 1.13 | 0.92 | 0, 48 | 35, 58 | 46 | EMEP |
| China | (0.99) | 0.89^a^ | - | 74, 15 | 137, 56 | 12 | World Data Centre for Greenhouse Gases (WDCGG), Carmichael *et al.* (2003), Huxiang *et al*. (2005), Li *et al*. (2007) |
| Northern India | (1.13) | - | - | 68, 20 | 90, 35 | 3 | Beig *et al*. (2007), Mittal *et al*. (2007), Engardt (2008), Debaje *et al*. (2009), Reddy *et a*l. (2008), Reddy *et al*. (2010) |
| Southern India | (1.23) | 1.04 | - | 68, 5 | 90, 19 | 3 | Ahammed *et al*. (2006), Beig *et al*. (2007), Mittal *et al*. (2007), Reddy *et al*. (2008), Roy *et al*. (2009), Debaje *et al*. (2010) |

^a^ AOT40 calculation based on five stations in April - June due to data availability

**Table S2**. Regionally-averaged ratios of modeled:observed O_3_ exposure according to daily mean O_3_ (M24), AOT40, and W126 (depending on data availability) during the wheat growing season in each region (Avnery *et al*., 2011). Data sources for observed O_3_, regional boundaries, and the number of observation stations per region are also listed. Observed AOT40 in China and India are from monitoring sites listed in Huixiang *et al*. (2005) and Roy *et al*. (2009), respectively.
