# Supplementary material for: Increasing global agricultural production by reducing ozone damages via methane emission controls and ozone-resistant cultivar selection
Source: Glob Chang Biol. 2013 Feb 5;19(4):1285–99. doi: 10.1111/gcb.12118 (PMC3627305; doi:10.1111/gcb.12118)
Supplement: Supplementary file 12 [file gcb0019-1285-SD11.docx]

|  | **Metric/CR Relationship** | **Crop Production (Mt)** | | | **%ΔCP (relative to 2000)** |
| --- | --- | --- | --- | --- | --- |
| **Country** |  | **CPL_CLE_** | **CPL_CH4-red_** | **ΔCP** |  |
| China | AOT40 – U.S./Europe | 39.0 | 34.7 | 4.3 | 4.5 |
|  | AOT40 - China | 58.3 | 51 | 7.3 | 7.6 |
| Japan | AOT40 – U.S./Europe | 0.24 | 0.21 | 0.03 | 4.8 |
|  | AOT40 - China | 0.57 | 0.47 | 0.10 | 19.1 |
| S. Korea | AOT40 – U.S./Europe | 0.0020 | 0.0018 | 0.0002 | 4.0 |
|  | AOT40 - China | 0.0033 | 0.0028 | 0.0005 | 10.0 |
| East Asia Total | AOT40 – U.S./Europe | 39.2 | 34.9 | 4.3 | 4.5 |
|  | AOT40 - China | 58.9 | 51.5 | 7.4 | 7.6 |

**Table S3**. Wheat crop production losses (CPL) and crop production (CP) improvements in East Asia due to methane mitigation estimated according to an AOT40 concentration:response (CR) function derived from Chinese cultivars of wheat (Wang *et al*., 2012), compared to mean sensitivity wheat CR functions based on U.S. and European field studies (Table S1).
