# Supplementary material for: Increasing global agricultural production by reducing ozone damages via methane emission controls and ozone-resistant cultivar selection
Source: Glob Chang Biol. 2013 Feb 5;19(4):1285–99. doi: 10.1111/gcb.12118 (PMC3627305; doi:10.1111/gcb.12118)
Supplement: Supplementary file 13 [file gcb0019-1285-SD12.docx]

|  | **Metric/CR Relationship** | **Crop Production (Mt)** | | | **%ΔCP (relative to 2000)** |
| --- | --- | --- | --- | --- | --- |
| **Country** |  | **CPL_CLE_** | **CPL_CH4-red_** | **ΔCP** |  |
| China | W126 - well watered median | 11.0 | 9.5 | 1.5 | 1.6 |
|  | W126 - droughted median | 2.3 | 1.9 | 0.4 | 0.4 |
| India | W126 - well watered median | 24.6 | 18.4 | 6.2 | 8.9 |
|  | W126 - droughted median | 7.9 | 5.4 | 2.5 | 3.6 |
| United States | W126 - well watered median | 2.6 | 2.3 | 0.3 | 0.5 |
|  | W126 - droughted median | 0.5 | 0.4 | 0.1 | 0.1 |
| World | W126 - well watered median | 60.3 | 46.8 | 13.5 | 2.4 |
|  | W126 - droughted median | 16.2 | 11.2 | 5.0 | 0.9 |

**Table S4**. Sensitivity study of calculated crop production loss (CPL) and crop production (CP) improvement due to CH_4_ mitigation according to all-crop, well-watered and droughted median CR functions (Table S1) as applied to wheat. These CPL estimates should not be literally interpreted, given that crop sensitivity to O_3_ greatly varies and wheat is more sensitive than the average all-crop response to ozone (Mills et al., 2007). These calculations assume 100% of wheat is rainfed and therefore either fully droughted or well-watered; in actuality, only ~60% of crops are rainfed and results should be scaled accordingly. See Supplemental Text for further discussion.
